# The Therapeutic Role of NPS-1034 in Pancreatic Ductal Adenocarcinoma as Monotherapy and in Combination with Chemotherapy

**DOI:** 10.3390/ijms25136919

**Published:** 2024-06-24

**Authors:** Yu-Ze Luan, Chi-Chih Wang, Chia-Ying Yu, Ya-Chuan Chang, Wen-Wei Sung, Ming-Chang Tsai

**Affiliations:** 1 School of Medicine, Chung Shan Medical University, Taichung 40201, Taiwan; s0901130@gm.csmu.edu.tw (Y.-Z.L.); cshy1192@csh.org.tw (C.-C.W.); cyyu2015@gmail.com (C.-Y.Y.); raptor7037@gmail.com (Y.-C.C.); 2 Division of Gastroenterology and Hepatology, Department of Internal Medicine, Chung Shan Medical University Hospital, Taichung 40201, Taiwan; 3 Institute of Medicine, Chung Shan Medical University, Taichung 40201, Taiwan; 4 Department of Urology, Chung Shan Medical University Hospital, Taichung 40201, Taiwan

**Keywords:** pancreatic cancer, NPS-1034, fluorouracil, oxaliplatin, synergistic effect

## Abstract

Pancreatic ductal adenocarcinoma (PDAC) poses a significant challenge in terms of diagnosis and treatment, with limited therapeutic options and a poor prognosis. This study explored the potential therapeutic role of NPS-1034, a kinase inhibitor targeting MET and AXL, in PDAC. The investigation included monotherapy with NPS-1034 and its combination with the commonly prescribed chemotherapy agents, fluorouracil and oxaliplatin. Our study revealed that NPS-1034 induces cell death and reduces the viability and clonogenicity of PDAC cells in a dose-dependent manner. Furthermore, NPS-1034 inhibits the migration of PDAC cells by suppressing MET/PI3K/AKT axis-induced epithelial-to-mesenchymal transition (EMT). The combination of NPS-1034 with fluorouracil or oxaliplatin demonstrated a synergistic effect, significantly reducing cell viability and inducing tumor cell apoptosis compared to monotherapies. Mechanistic insights provided by next-generation sequencing indicated that NPS-1034 modulates immune responses by inducing type I interferon and tumor necrosis factor production in PDAC cells. This suggests a broader role for NPS-1034 beyond MET and AXL inhibition, positioning it as a potential immunity modulator. Overall, these findings highlight the anticancer potential of NPS-1034 in PDAC treatment in vitro, both as a monotherapy and in combination with traditional chemotherapy, offering a promising avenue for further in vivo investigation before clinical exploration.

## 1. Introduction

Pancreatic cancer is a deadly disease, and pancreatic ductal adenocarcinoma (PDAC) is the most prevalent and deadly type of pancreatic cancer. According to GLOBOCAN 2020, the age-standardized incidence and mortality rates for pancreatic cancer are 4.9 and 4.5 people (per 100,000 population), respectively, and it causes the eighth highest number of deaths among all cancer types [1,2]. Apart from the high mortality rate, managing pancreatic cancer is problematic. Due to the difficulty of early diagnosis, a mere 15–20% of pancreatic cancer cases are resectable. However, surgery is the only treatment that can provide a cure, and the postoperative prognosis is still poor, with a high recurrence rate [2,3]. Regarding the 80–85% of pancreatic cancers that are unresectable, systemic therapies are commonly prescribed as follows: dual therapy with gemcitabine and nab-paclitaxel; quadruple therapy with folinic acid, fluorouracil, irinotecan, and oxaliplatin (FOLFIRINOX); and monotherapy with S-1. However, their prognoses are poor, with the median overall survival being less than 25 months at the maximum [4,5,6,7,8]. Therefore, there is an urgent demand for research on pancreatic cancer treatment [9].

Clinically, elevated expression of MET proto-oncogene, receptor tyrosine kinase (MET) and AXL receptor tyrosine kinase (AXL) or their ligands correlates with worse status in pancreatic cancer patients [10,11]. MET and AXL are important regulators of pancreatic cancer because they are receptor tyrosine kinases (RTKs) that function normally in healthy conditions. For example, MET can facilitate wound repair, and AXL can regulate cell survival [12,13]. However, their aberrant expression contributes to the progression of various types of cancer, including pancreatic cancer. Downstream reactions to these aberrant expressions are related to immune suppression, tumor cell survival, motility, angiogenesis, epithelial-to-mesenchymal transition (EMT), metastasis, and chemoresistance [13,14,15,16]. Hence, the dual inhibition of MET and AXL could be promising for pancreatic cancer treatment.

NPS-1034 is an inhibitor to kinases including AXL, MET, and a variety of MET mutants [17]. MET mutants are related to tumor formation and metastasis in many kinds of cancers, and NPS-1034 inhibits not only wild-type MET but also many MET mutants. Importantly, NPS-1034 can inhibit three MET mutants that three MET inhibitors—SU11274, PHA665752, and NVP-BVU972—cannot inhibit. These effects indicate its anticancer potential. A xenograft experiment on gastric cancer also revealed that the anti-angiogenetic and pro-apoptotic effects of NPS-1034 decreased tumor growth without virtual weight loss in mice [18]. In studies on non-small cell lung cancer (NSCLC) cells resistant to epidermal growth factor receptor tyrosine kinase inhibitor (EGFR-TKI), NPS-1034 effectively overcame drug resistance and reduced the viabilities of drug-resistant cells in vitro via AXL or MET inhibition. Consistent with this evidence, dual therapy with NPS-1034 and EGFR-TKI can also prevent or reduce the regrowth of NSCLC tumors in vivo via antiproliferation and apoptosis without apparent adverse effects or weight loss [17,19].

NPS-1034 has shown positive effects on several types of cancers, including gastric cancer and NSCLC [18,19]. However, despite the important roles that MET and AXL play in the progression of pancreatic cancer, there has been no research on NPS-1034 and pancreatic cancer. We hypothesize that NPS-1034 possesses anticancer effects on pancreatic cancer. Therefore, this study aimed to investigate these effects and their underlying mechanisms through in vitro experiments. The synergistic effect of NPS-1034 with commonly prescribed chemotherapy agents for pancreatic cancer—fluorouracil and oxaliplatin—was also tested.

## 2. Results

### 2.1. NPS-1034-Induced Cell Death Decreased the Viability and Clonogenicity of PDAC Cells

**Figure 1:**
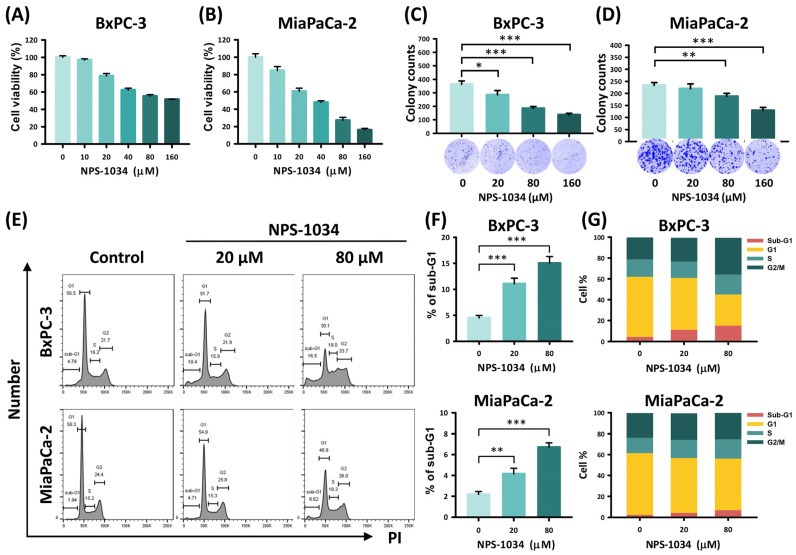
NPS-1034 caused cell death of PDAC cells. (**A**,**B**) The MTT assay showed reduced PDAC cell viability after NPS-1034 treatment. (**C**,**D**) The colony formation assay demonstrated a decline in colony formation after NPS-1034 treatment on PDAC cells. (**E**) Flow cytometry revealed increasing proportions of the sub-G1 group after NPS-1034 treatment on PDAC cells. (**F**) The columns illustrate the increasing proportions of the sub-G1 group after NPS-1034 treatment on PDAC cells. (**G**) Cell cycle distributions of PDAC cells after NPS-1034 treatment are shown. Data are shown as mean ± SD (* *p* < 0.05; ** *p* < 0.01; *** *p* < 0.001).

The influence of NPS-1034 on the growth of human PDAC cells, including BxPC-3 and MIA PaCa-2 cells, was tested using MTT and clonogenic assays. As shown in Figure 1A,B, the MTT assay was performed to test the impact of NPS-1034 on the viability of PDAC cells. It is shown that the viabilities of PDAC cells decreased dose-dependently after a 1-day treatment with 0, 10, 20, 40, 80, and 160 μM NPS-1034. As for clonogenicity, Figure 1C,D show that the colony formation of PDAC cells decreased dose-dependently after treatment with 0, 20, 80, and 160 μM NPS-1034 for 1 day and incubation for 9 days. The number of BxPC-3 colonies decreased from 364.0 ± 24.3 (control group) to 285.3 ± 31.5 (20 μM group), 184.7 ± 14.0 (80 μM group), and 137.0 ± 10.8 (160 μM group). The MIA PaCa-2 colonies decreased from 233.7 ± 11.5 (control group) to 219.7 ± 20.1 (20 μM group), 187.7 ± 12.7 (80 μM group), and 129.3 ± 12.5 (160 μM group) (BxPC-3: control vs. 20 μM, 80 μM, and 160 μM, *p*-value = 0.027, <0.001, and <0.001, respectively; MIA PaCa-2: control vs. 20 μM, 80 μM, and 160 μM, *p*-value = 0.354, <0.01, and <0.001, respectively). In summary, the viability and clonogenicity of PDAC cells were curbed by NPS-1034 in a dose-dependent manner.

To determine the cause of these effects, a cell cycle analysis was performed. Figure 1E–G illustrate the dose-dependent increase in the sub-G1 group of PDAC cells after 48 h of treatment with 0, 20, and 80 μM NPS-1034 (BxPC-3: control vs. 20 μM and 80 μM, all *p*-values < 0.001; MIA PaCa-2: control vs. 20 μM and 80 μM, *p*-value = 0.006 and <0.001, respectively). The proportion of BxPC-3 cells in the sub-G1 phase grew from 4.48% ± 0.49% (control group) to 11.07% ± 1.07% (20 μM group) and 15% ± 1.31% (80 μM group). The proportion of MIA PaCa-2 cells in the sub-G1 phase grew from 2.17% ± 0.28% (control group) to 4.13% ± 0.55% (20 μM group) and 6.70% ± 0.44% (80 μM group). This indicates that NPS-1034 causes PDAC cell death.

### 2.2. Apoptosis Is the Major Type of Cell Death in PDAC Cells Treated with NPS-1034

**Figure 2:**
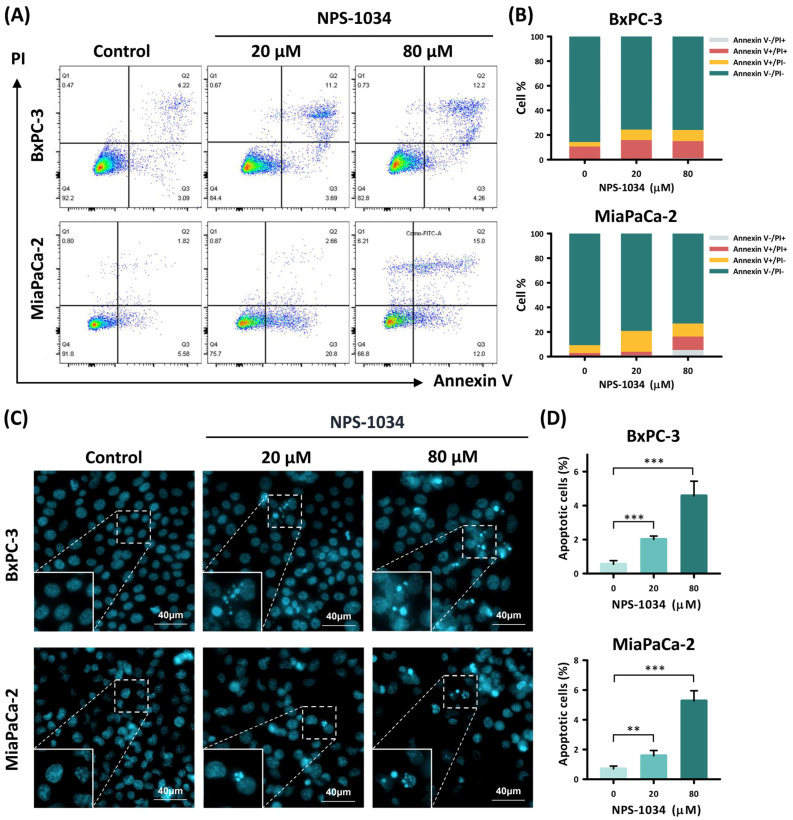
NPS-1034 triggered apoptosis in PDAC cells. (**A**) Flow cytometry of Annexin V/PI double staining revealed distributions of early apoptosis, late apoptosis, and necrosis groups of PDAC cells with NPS-1034 treatment. (**B**) The columns demonstrate the proportions of Annexin V+/−/PI+/− PDAC cells with NPS-1034 treatment. (**C**) Hoechst 33342 staining shows morphological changes and an increased proportion of apoptotic cells in PDAC cells after NPS-1034 treatment. (**D**) The bar chart illustrates an increasing proportion of apoptotic PDAC cells after NPS-1034 treatment. Data are shown as mean ± SD (** *p* < 0.01; *** *p* < 0.001).

We further investigated the types of cell death occurring in NPS-1034-treated PDAC cells through Annexin V/PI double staining and Hoechst 33342 staining (Figure 2). For Annexin V/PI double staining (Figure 2A,B), we conducted a 48-hour treatment with 0, 20, and 80 μM NPS-1034 on PDAC cells. The proportion of early apoptotic (Annexin V+/PI−) and late apoptotic (Annexin V+/PI+) PDAC cells increased with NPS-1034 treatment compared to the control groups. The proportion of apoptotic BxPC-3 cells grew from 13.57% ± 1.35% (control group) to 23.70% ± 4.93% (20 μM group) and 22.95% ± 1.57% (80 μM group). The proportion of apoptotic MIA PaCa-2 cells grew from 8.45% ± 1.63% (control group) to 19.83% ± 3.29% (20 μM group) and 21.51% ± 7.38% (80 μM group) (BxPC-3: control vs. 20 μM and 80 μM, *p*-value = 0.027 and <0.001, respectively; MIA PaCa-2: control vs. 20 μM and 80 μM, *p*-value = 0.006 and 0.04, respectively).

For Hoechst 33342 staining (Figure 2C,D), we conducted a 48-hour treatment with 0, 20, and 80 μM NPS-1034 on PDAC cells. The results were consistent with those of Annexin V/PI double staining. The proportions of apoptotic PDAC cells increased dose-dependently with NPS-1034 treatment compared to the control groups (BxPC-3: control vs. 20 μM and 80 μM, all *p*-values < 0.001; MIA PaCa-2: control vs. 20 μM and 80 μM, *p*-value = 0.009 and <0.001, respectively). The proportion of apoptotic BxPC-3 cells grew from 0.55% ± 0.21% (control group) to 2.01% ± 0.20% (20 μM group) and 4.57% ± 0.86% (80 μM group). The proportion of apoptotic MIA PaCa-2 cells grew from 0.70% ± 0.19% (control group) to 1.58% ± 0.35% (20 μM group) and 5.27% ± 0.69% (80 μM group) (BxPC-3: control vs. 20 μM and 80 μM, all *p*-values < 0.001; MIA PaCa-2: control vs. 20 μM and 80 μM, *p*-value = 0.009 and <0.001, respectively).

### 2.3. A 24-Hour NPS-1034 Treatment Suppressed the Migration of PDAC Cells via the Inhibition of MET-Induced EMT

**Figure 3:**
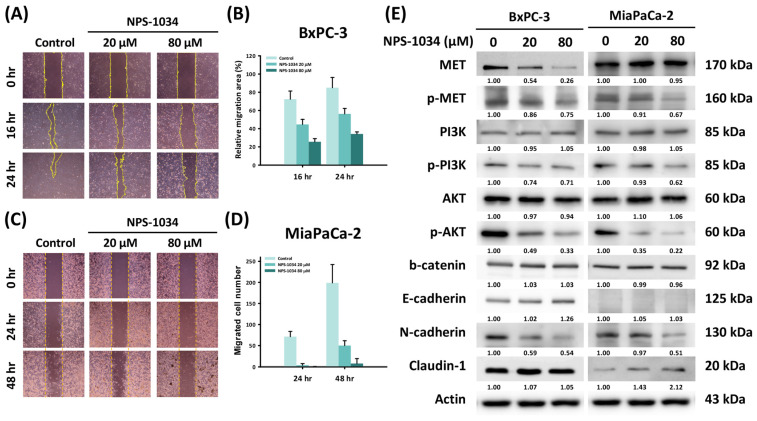
NPS-1034 suppressed the migration of PDAC cells. (**A**,**C**) The wound healing assay revealed the migration of PDAC cells with NPS-1034 treatment. The yellow lines indicate the margins for analysis. (**B**,**D**) The columns demonstrate the proportions of migrated PDAC cells with NPS-1034 treatment. (**E**) Western blot investigation of proteins related to the MET-induced EMT pathway. Data are shown as mean ± SD.

To investigate the influence of NPS-1034 on the migration of PDAC cells, a wound healing assay was performed on PDAC cells treated with 0, 20, and 80 μM NPS-1034 for 0, 16, and 24 h (BxPC-3 cells) or for 0, 24, and 48 h (MIA PaCa-2 cells). As shown in Figure 3A,B, the percentage of the relative migration area of BxPC-3 cells with a 16-hour treatment decreased from 72.38% ± 9.01% (control) to 44.65% ± 5.58% (20 μM) and 25.6% ± 3.48% (80 μM). For BxPC-3 cells with a 24-hour treatment, it decreased from 85.02% ± 11.21% (control) to 56.28% ± 5.99% (20 μM) and 34.2% ± 2.25% (80 μM). As seen in Figure 3C,D, the number of migrated MIA PaCa-2 cells with a 24-hour treatment decreased from 71.25 ± 12.34 (control) to 3.67 ± 3.67 (20 μM) and 0.17 ± 0.41 (80 μM). For MIA PaCa-2 cells with a 48-hour treatment, it decreased from 198.75 ± 43.57 (control) to 50 ± 11.51 (20 μM) and 7.5 ± 11.66 (80 μM) (BxPC-3: 16 h, control vs. 20 μM and 80 μM, all *p*-values < 0.001; 24 h, control vs. 20 μM and 80 μM, all *p*-values < 0.001; MIA PaCa-2: 24 h, control vs. 20 μM and 80 μM, all *p*-values < 0.001; 48 h, control vs. 20 μM and 80 μM, all *p*-values < 0.001).

To further understand the mechanism behind this antimigratory effect of NPS-1034 on PDAC cells, Western blotting was performed on PDAC cells treated with 0, 20, and 80 μM NPS-1034 for 24 h. We tested the proteins of the MET-induced EMT pathway and observed dose-dependent inhibition of MET phosphorylation and its downstream pathway through downregulation of phosphorylated MET, phosphorylated phosphoinositide 3-kinase (PI3K), and phosphorylated Akt. As for following the EMT pathway, we also observed dose-dependent inhibition through upregulation of epithelial biomarkers, including E-cadherin in BxPC-3 cells and claudin-1 in MIA PaCa-2 cells, and through downregulation of the mesenchymal biomarker N-cadherin. Therefore, from the above results, we suggest that even though 24-hour treatment with NPS-1034 did not significantly induce the apoptosis of PDAC cells, it successfully suppressed the migration of PDAC cells by inhibiting MET phosphorylation and its downstream EMT pathway.

### 2.4. The Synergistic Effect of NPS-1034 and Common Anti-PDAC Drugs (Fluorouracil or Oxaliplatin) Was Found in PDAC Cells after 24 h of Combined Treatment

Although the above experiments have shown the success of NPS-1034 treatment in apoptosis induction and the suppression of migration in PDAC cells, the 24-hour treatment seems less effective in apoptosis induction. Therefore, we considered the effect of combined treatment with NPS-1034 and the commonly prescribed anti-PDAC chemotherapeutic drugs fluorouracil and oxaliplatin. MTT assays were conducted to investigate the viabilities of PDAC cells under monotherapy with NPS-1034 (25, 50, and 100 μM), fluorouracil (10, 20, and 40 μM), and oxaliplatin (5, 10, and 20 μM) and dual therapy with NPS-1034 + fluorouracil and NPS-1034 + oxaliplatin. These cells were cultivated for 24 h and then treated with the drugs for 24 h.

**Figure 4:**
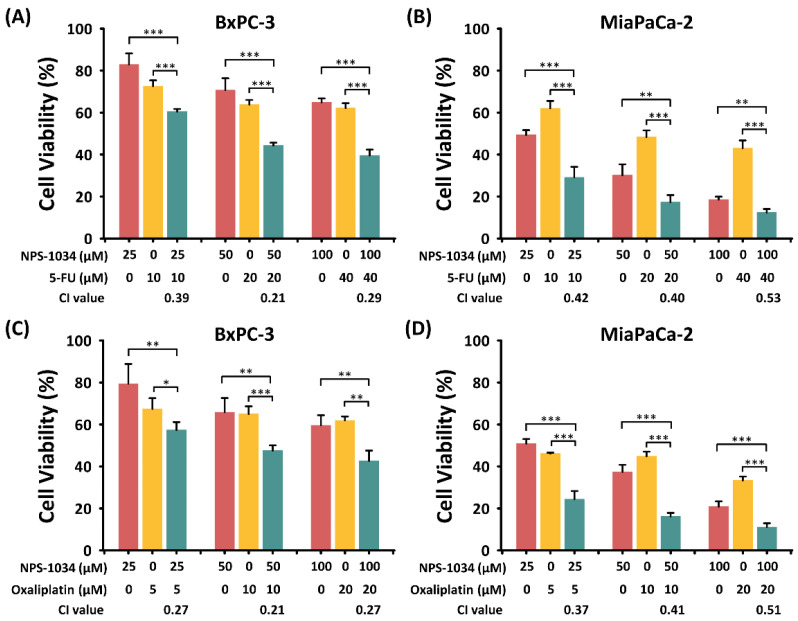
The viability of PDAC cells reduced more drastically after combined treatment with NPS-1034 + 5-fu/oxaliplatin. (**A**,**B**) MTT assay showing reduced viability of PDAC cells after treatment with NPS-1034 + fluorouracil (5-FU). (**C**,**D**) MTT assay showing reduced viability of PDAC cells after treatment with NPS-1034 + oxaliplatin. Data are shown as mean ± SD (* *p* < 0.05; ** *p* < 0.01; *** *p* < 0.001).

As shown in Figure 4, cell viability was significantly lower with dual therapy than with monotherapy with NPS-1034, fluorouracil, and oxaliplatin (Figure 4A–D). Additionally, dual therapy caused a dose-dependent decrease in the viability of PDAC cells (Figure 4A–D). Clearly, the combination of NPS-1034 with commonly prescribed anti-PDAC drugs has a significant synergistic effect of suppressing the viability of PDAC cells. Through this experiment, we can further understand the potential of NPS-1034 in clinical use against PDAC.

### 2.5. Apoptosis Was Triggered by the Synergistic Effect of Combined Treatment with NPS-1034 and Common Anti-PDAC Drugs (Fluorouracil or Oxaliplatin)

**Figure 5:**
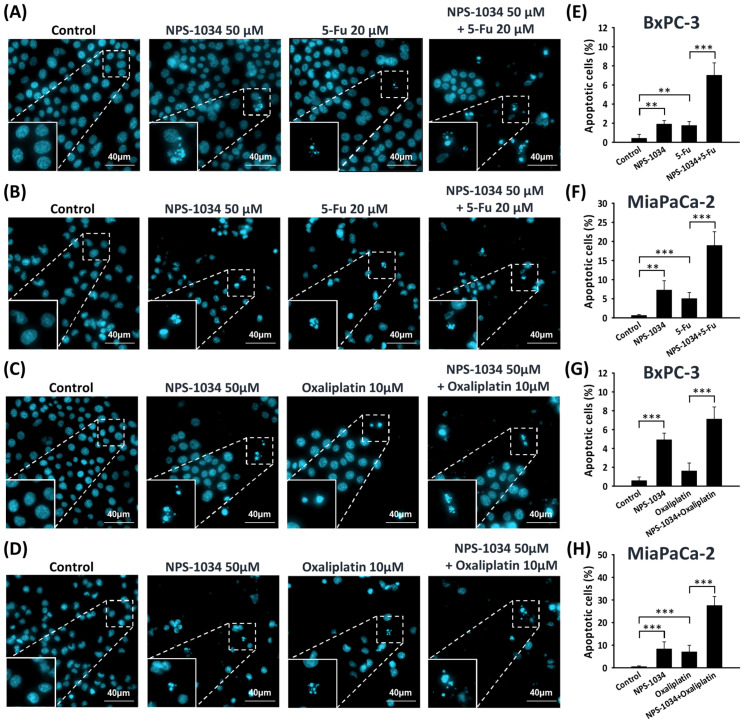
Combined treatment with NPS-1034 triggered apoptosis in PDAC cells. (**A**–**D**) Hoechst 33342 staining showing morphological changes and an increase in the proportion of apoptotic cells in PDAC cells after treatment with NPS-1034, fluorouracil, oxaliplatin, and their combined therapies. (**E**–**H**) The bar chart illustrates the increasing proportions of apoptotic PDAC cells after these treatments. Data are shown as mean ± SD (** *p* < 0.01; *** *p* < 0.001).

To further understand the pro-apoptotic effect of the combined therapy of NPS-1034 and common anti-PDAC drugs (fluorouracil or oxaliplatin), Hoechst 33342 staining was conducted. This testing showed that NPS-1034 causes similar or higher apoptotic rates compared with common anti-PDAC drugs, and both monotherapies significantly induce apoptosis in PDAC cells (Figure 5A,E: control vs. NPS-1034 and control vs. fluorouracil, *p*-value = 0.004 and 0.003, respectively; Figure 5B,F: control vs. NPS-1034 and control vs. fluorouracil, *p*-value = 0.002 and <0.001, respectively; Figure 5C,G: control vs. NPS-1034 and control vs. oxaliplatin, *p*-value < 0.001 and =0.087, respectively; Figure 5D,H: control vs. NPS-1034 and control vs. oxaliplatin, all *p*-values < 0.001). Additionally, the combined therapy of NPS-1034 and common anti-PDAC drugs showed significantly higher apoptotic rates than the monotherapies of common anti-PDAC cells (BxPC-3: fluorouracil vs. NPS-1034 + fluorouracil, oxaliplatin vs. NPS-1034 + oxaliplatin, all *p*-values < 0.001; MIA PaCa-2: fluorouracil vs. NPS-1034 + fluorouracil, oxaliplatin vs. NPS-1034 + oxaliplatin, all *p*-values < 0.001). These findings suggest that NPS-1034 treatment as well as the combination of NPS-1034 and common anti-PDAC drugs both cause a higher apoptotic rate than monotherapy with the common anti-PDAC drugs fluorouracil and oxaliplatin.

### 2.6. Next-Generation Sequencing Showed That NPS-1034 Can Modulate Immune Responses by Inducing Type I Interferon and Tumor Necrosis Factor Production in PDAC Cells

**Figure 6:**
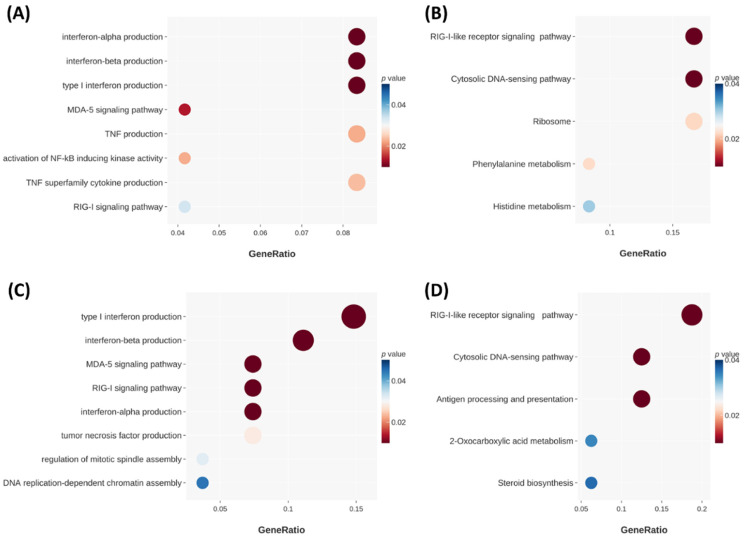
Next-generation sequencing (NGS) with the Gene Oncology (GO) biological process and Kyoto Encyclopedia of Genes and Genomes (KEGG) databases revealed a change in gene expression between PDAC cells treated with fluorouracil/oxaliplatin and fluorouracil/oxaliplatin + NPS-1034. The charts show the gene groups that account for the greatest proportions of changed genes and are sequenced in the order of their proportions. These data were analyzed by combining NGS results from both cell lines. (**A**) Results of fluorouracil compared to fluorouracil + NPS-1034 treatment with the GO database. (**B**) Results of fluorouracil compared to fluorouracil + NPS-1034 treatment with the KEGG database. (**C**) Results of oxaliplatin compared to oxaliplatin + NPS-1034 treatment with the GO database. (**D**) Results of oxaliplatin treatment compared to oxaliplatin + NPS-1034 treatment with the KEGG database.

To comprehend the mechanism behind the increase in apoptotic PDAC cells after treatment with NPS-1034 + fluorouracil/oxaliplatin versus those treated only with fluorouracil or oxaliplatin, NGS was conducted with the GO and KEGG databases. There were 47 genes significantly altered when comparing fluorouracil alone to fluorouracil combined with NPS-1034 (31 genes upregulated and 16 genes downregulated). Additionally, 51 genes were significantly altered when comparing oxaliplatin alone to oxaliplatin combined with NPS-1034 (17 genes upregulated and 34 genes downregulated). The charts show the gene groups that accounted for the greatest proportion of genes with differential expressions. In Figure 6A, the genes expressed by PDAC cells treated with 20 μM fluorouracil and 20 μM fluorouracil + 50 μM NPS-1034 are compared and categorized with GO terms regarding the biological process. Interferon (IFN) alpha production changed most significantly, followed by IFN beta production, type I IFN production, the MDA-5 signaling pathway, tumor necrosis factor (TNF) production, activation of NFκB-inducing kinase activity, TNF superfamily cytokine production, and the RIG-I signaling pathway, as indicated by the *p*-values. In Figure 6B, the genes expressed by PDAC cells treated with 20 μM fluorouracil and 20 μM fluorouracil + 50 μM NPS-1034 are compared and categorized using the KEGG pathways. The RIG-I signaling pathway accounted for the greatest proportions, followed by the cytosolic–DNA sensing pathway, ribosome, phenylalanine metabolism, and histidine metabolism.

As for oxaliplatin treatment, as shown in Figure 6C, the genes expressed by PDAC cells treated with 10 μM oxaliplatin and 10 μM oxaliplatin + 50 μM NPS-1034 were compared and categorized with GO terms regarding the biological process. Type I IFN production changed most significantly, followed by IFN beta production, the MDA-5 signaling pathway, the RIG-I signaling pathway, IFN alpha production, TNF production, regulation of mitotic spindle assembly, and DNA replication-dependent chromatin assembly, as indicated by the *p*-values. As shown in Figure 6D, the genes expressed by PDAC cells treated with 10 μM oxaliplatin and 10 μM oxaliplatin + 50 μM NPS-1034 were compared and categorized using the KEGG pathways. The RIG-I-like signaling pathway accounted for the greatest proportion, followed by the cytosolic DNA-sensing pathway, antigen processing and presentation, 2-oxocarboxylic acid metabolism, and steroid biosynthesis.

These figures suggest that NPS-1034 induced the following reactions: (1) The immune response was triggered in PDAC cells, including IFN production, TNF production, and antigen processing and presentation. These may be the downstream reactions of cytosolic DNA sensing, RIG-I signaling, MDA-5 signaling, and the NFκB pathway. (2) Cell cycle arrest was induced, as revealed by the change in genes regarding the assembly of the mitotic spindle and DNA replication-dependent chromatin. They might be induced by IFN and TNF. (3) The fundamental metabolisms of PDAC cells were also influenced, including genes related to ribosome, phenylalanine, 2-oxocarboxylic acid, histidine, and steroid biosynthesis. This could be the aftermath of the abovementioned events induced by NPS-1034 and could undermine the basic functioning of PDAC cells.

In conclusion, NGS not only explains the possible mechanisms behind the apoptosis of PDAC cells but also shows us other potential avenues for NPS-1034 as a treatment. NPS-1034 is more than an inhibitor of MET and AXL; it could be an immune modulator by inducing IFN and TNF. Therefore, we are convinced that the combination of NPS-1034 with commonly prescribed anti-PDAC chemotherapies sheds new light on PDAC treatment.

## 3. Discussion

This study aimed to investigate the effect of NPS-1034 treatment on pancreatic cancer. Therefore, we experimented with the most prevalent and deadly type of pancreatic cancer—PDAC. The findings show that NPS-1034 causes cell death in BxPC-3 and MIA PaCa-2 cells (PDAC cells), and that the cell death is mainly apoptosis. Notably, NPS-1034 suppresses the migration of PDAC cells, and it is the inhibition of MET-induced EMT that contributes to the antimigratory effect. Additionally, when its effect on cell viability and apoptosis was compared with commonly prescribed anti-PDAC chemotherapies—fluorouracil and oxaliplatin—we found that the combined therapy of NPS-1034 with fluorouracil or oxaliplatin caused significantly higher rates of viability suppression and apoptosis induction than monotherapy with NPS-1034, fluorouracil, or oxaliplatin. Finally, NGS was carried out to understand the full structure of the mechanisms behind the effect of NPS-1034 on PDAC cells at the genetic level. It was found that the pro-apoptotic and antimigratory effects of NPS-1034 may result from type I IFN and TNF induced by the RIG-I and MDA-5 signaling pathways. This is the first study to reveal the impact of NPS-1034 on cancer migration and the mechanism involved. This is also the first study of the effect of NPS-1034, a dual inhibitor of MET and AXL, on pancreatic cancer. This cancer highly expresses MET and AXL, and the combination of NPS-1034 with the commonly prescribed anti-PDAC chemotherapies oxaliplatin and fluorouracil shows a remarkably effective synergistic effect. Finally, this is the first study to reveal that NPS-1034 is an IFN and TNF inducer, and the mechanism of apoptosis and inhibition of migration could also be triggered by this NPS-1034-induced immune response.

Our findings demonstrated that 48 h of treatment with NPS-1034 caused PDAC cell death and therefore decreased their viability and clonogenicity. The cell death involved was mainly apoptosis. This cytotoxic effect of NPS-1034 on cancer cells has also been reported in other cancers. In an NSCLC model, NPS-1034 inhibited ROS1 activity and cell proliferation in HCC78 cells [17]. In the testicular cancer cell lines NCCIT and NTERA2, NPS-1034 caused apoptosis, leading to declines in viability and clonogenicity [20]. In gastric cancer cells, NPS-1034 inhibited the viability and clonogenicity of the high MET-expressing cell lines MKN45 and SNU638 and induced apoptosis in MKN45 cells. Additionally, an in vivo study reaffirmed NPS-1034’s anticancer effect via the inhibition of MKN45 xenograft tumor growth in nude mice via its anti-angiogenetic and pro-apoptotic properties [18]. Our study proved that NPS-1034 also promotes apoptosis of PDAC cells and subsequently reduces viability and clonogenicity. As for its mechanism, we also performed a human apoptosis array, but no significant change in protein expression was observed between 0 and 80 μM NPS-1034-treated PDAC cells (Supplementary Figure S1). This discrepancy might be due to the small panel of proteins included in the array. However, significant changes in gene expression related to apoptosis were observed with NGS between treatment with fluorouracil/oxaliplatin and fluorouracil/oxaliplatin + NPS-1034, which is described in detail below.

In PDAC patients, pancreatic stellate cells (PSCs) produce hepatocyte growth factor (HGF) and pancreatic cancer cells express MET. Their binding causes downstream responses that trigger migration, invasion, and proliferation, which are possibly related to tumor cell motility [14]. It has been shown that serum HGF expression is higher in patients with invasive pancreatic cancer, and overexpression of MET is related to more severe invasion into lymph nodes as well as shorter survival, higher recurrence rates, and later stages of tumors [21,22]. Therefore, we wanted to explore the antimigratory effect of NPS-1034, a dual inhibitor of MET and AXL, on PDAC cells.

To investigate the influence of NPS-1034 on migration, we carried out a series of experiments on 24-hour NPS-1034-treated PDAC cells. In addition to the wound healing assay and Western blotting, we performed cell cycle analysis on PDAC cells with 24-hour treatment with 0, 20, and 80 μM NPS-1034. There were dose-dependent increases in the sub-G1 groups, but the increase did not reach statistical significance. Although the 24-hour NPS-1034 treatment for PDAC cells did not show a significant effect on apoptosis induction for either cell line, it showed a potent antimigratory effect. It not only successfully decreased the migration of PDAC cells but also inhibited the downstream EMT of the MET/PI3K/Akt pathway. Previous studies have yielded similar findings. For the human pancreatic cancer cell line AsPC-1, Pothula et al. proved that the inhibition of HGF decreased the occurrence of tumor metastasis in a mouse model [23]. A study of the human pancreatic cancer cell lines SW1990 and PANC-1 [15] suggested that the binding of HGF and MET causes EMT-associated chemoresistance through its downstream PI3K/Akt pathway. The study also suggested that PSC downregulates gemcitabine-induced cleavage of caspase 8 and caspase 3, which can be eliminated by an MET inhibitor. The above studies revealed that the binding of HGF and MET is associated with EMT, tumor metastasis, and anti-apoptosis in human pancreatic cancer cell lines. Our study not only proved that this effect also presents in BxPC-3 and MIA PaCa-2 cells but also showed that the inhibition of MET is capable of inhibiting EMT and thus decreasing the migration of PDAC cells.

Although both the BxPC-3 and MIA PaCa-2 cell lines showed dose-dependent inhibition of EMT, there were some differences in the expression of EMT-related proteins between the two cell lines. In both cell lines, we observed a decrease in phosphorylated MET, phosphorylated PI3K, and phosphorylated Akt, indicating the inhibition of the MET/PI3K/Akt pathway by NPS-1034. In BxPC-3 cells, we observed upregulation of E-cadherin (the epithelial marker) and downregulation of N-cadherin (the mesenchymal marker). In MIA PaCa-2 cells, although we could not observe the expression of E-cadherin, upregulation of another epithelial marker, claudin-1, and downregulation of N-cadherin were observed. In 2011, a study on pancreatic cancer cells and E-cadherin showed that MIA PaCa-2 cells do not express E-cadherin naturally [24]. Another study that researched the invasion of pancreatic cancer cell lines also demonstrated this effect. Only four of the eight PDAC cell lines tested in the study expressed E-cadherin [25]. In summary, despite the absence of E-cadherin expression in MIA PaCa-2 cells, our findings demonstrate that the inhibition of MET by NPS-1034 successfully inhibits the migration of PDAC cells via EMT induced by the MET/PI3K/Akt pathway.

The ability of PDAC cells to migrate is closely associated with the metastasis of PDAC, which is crucial to the prognosis of patients with PDAC. The 5-year relative survival rate for patients with localized stage (confined to primary site) pancreatic cancer at diagnosis is 43.9%. However, that for patients with regional stage cancer (spread to regional lymph nodes) is 14.7%, and that for the distant stage (cancer has metastasized) is only 3.1%. Moreover, 50% of pancreatic cancer patients have distant stage disease, while only 10% to 15% have localized stage cancer [26]. Therefore, a potent inhibitor of the migration of PDAC cells, such as NPS-1034, could be an important key to PDAC treatment.

Although metastasis is a significant issue in PDAC, treatment against it remains insufficient. Apart from traditional antimetastasis strategies for PDAC (i.e., killing cancer stem cells), there are new strategies that include targeting circulating tumor cells and tumor microenvironments via nanomedicine; nevertheless, the evidence is still weak [27]. Our study contributes to the development of a strategy for blocking the MET pathway. Many MET inhibitors are now being researched for the treatment of pancreatic cancer. Although some show flaws, such as inevitable chemoresistance in patients, some seem effective and are part of current clinical trials [14]. Therefore, we believe that NPS-1034 has the potential to treat PDAC, and our findings could strengthen this idea. We showed that the dual inhibitor of MET and Axl, NPS-1034, not only inhibits PDAC cell migration by blocking MET and its downstream EMT but also induces apoptotic cell death and thus reduces the viability and clonogenicity of PDAC cells.

To realize the clinical applicability and strengthen the pro-apoptotic ability of NPS-1034 treatment for PDAC, we researched combined treatment with NPS-1034 and the commonly prescribed chemotherapy agents fluorouracil and oxaliplatin. The dual treatment showed significantly higher anti-viability and pro-apoptosis effects on PDAC cells. This result shows that the combination of NPS-1034 with either of the current chemotherapy agents for PDAC may greatly improve the condition of PDAC patients. Therefore, we conducted NGS to compare the gene expression of PDAC cells under combined treatment versus only fluorouracil or oxaliplatin.

Supplementary Figure S2 shows the results from NPS-1034 monotherapy. The following conclusions were drawn from the results of combination therapy according to the GO and KEGG databases: (1) NPS-1034 is not merely an MET and AXL inhibitor but also an IFN and TNF inducer. Gene expression related to immune pathways changed, making it highly possible that NPS-1034 was responsible for the apoptosis of PDAC cells, including cytosolic DNA sensing, antigen processing and presentation, RIG-I and MDA-5 signaling, IFN production, the NFκB pathway, and the TNF pathway. Studies have shown that the RIG-I and MDA-5 signaling pathways induce not only type I IFN production but also the NFκB pathway, leading to TNF production [28,29]. Subsequently, TNF and type I IFN could inhibit proliferation, arrest the cell cycle, and trigger apoptosis in PDAC cells [30,31]. Despite the controversy regarding the mechanism behind this, it is widely accepted that type I IFN inhibits tumor metastasis and invasion, and some studies have shown that EMT is the cause. Thus, type I IFN may also account for our findings regarding the antimetastatic effect of NPS-1034 [32,33]. As for the change in the cytosolic DNA sensing pathway, it may induce not only the RIG-I pathway but also the antigen processing and presentation pathway. The antigen processing and presentation pathway may also be reinforced by IFN and TNF forming the RIG-I pathway and activating natural killer cells and T cells, making NPS-1034 an even more powerful anticancer agent in vivo [34,35]. Future in vivo experiments are needed to examine this effect. (2) Gene expression in terms of the cell cycle changed, including mitotic spindle regulation and DNA replication-dependent chromatin assembly. This could impair the proliferation and DNA expression of PDAC cells [36,37]. These changes could also correspond to the abovementioned IFN production. Research has shown that IFN is related to cell cycle arrest in cancer cells [38]. (3) Some relationships to lipid and protein metabolism changed, including ribosome, steroid, histidine, phenylalanine, and 2-oxocarboxylic acid metabolism. These changes in fundamental metabolism may be a reaction to all of the above effects of PDAC cells or may undermine the ability of PDAC cells to survive, proliferate, migrate, etc.

IFN’s ability to induce apoptosis and cell cycle arrest in PDAC cells has been proven [39]. The positive effect of combining IFN and chemotherapies in pancreatic cancer has also been proven in vivo and in clinical studies, including in combination with gemcitabine, fluorouracil, and cisplatin [40,41]. However, an old phase 2 clinical trial showed that the toxicity of fluorouracil + IFN alpha was too high to be applied in pancreatic cancer patients [42]. Therefore, future in vivo studies of chemotherapies combined with NPS-1034 in pancreatic cancer are needed to acknowledge its toxicity and applicability. We hope that this novel drug will be beneficial to numerous patients suffering from this deadly disease.

There are some limitations to our study. First, only specific cell lines were tested. The BxPC-3 and MIA PaCa-2 cell lines cannot fully represent all types of PDAC. Secondly, we did not independently verify the specific kinase inhibition role of NPS-1034 on MET and AXL. Thirdly, only in vitro experiments were performed in this study. Due to the limited sample size in RNA sequencing, we could only use *p*-values instead of adjusted *p*-values for the analysis. The lack of in vivo studies can lead to ignorance of the adverse effects of NPS-1034 in PDAC treatment. The influence of significantly changed immune-related pathways on the tumor microenvironment remains unknown. Therefore, further research on other PDAC cell lines and animal models is needed to ascertain the effects of NPS-1034 treatment on PDAC.

## 4. Materials and Methods

### 4.1. Cell Culture

BxPC-3 and MIA PaCa-2, human PDAC cells, were purchased from the JCRB cell bank (Ibaraki, Osaka, Japan) and kept in accordance with the supplier’s instructions. These cells were incubated at 37 °C with 5% CO_2_ in RPMI medium with 10% fetal bovine serum, 2 g/mL NaHCO_3_, 100 U/mL penicillin, 100 μg/mL streptomycin, 1 mM sodium pyruvate, and 0.1 mM non-essential amino acids.

### 4.2. MTT Assays

MTT assays were applied to test the cytotoxicity of NPS-1034. In brief, we seeded 10^4^ cells into 96-well plates, incubated them overnight, and exposed them to NPS-1034 (0, 20, 40, 80, and 160 μM) for 24 h. Then, we added MTT solution (0.5 mg/mL) to the wells, incubated them for 3 h at 37 °C, removed the supernatant, and dissolved the formazan product in DMSO. Finally, an ELISA reader was utilized to measure the optical density with a 570 nm filter. These experiments were conducted in triplicate, and the cell viabilities were calculated and analyzed by comparing them to the control group.

### 4.3. Clonogenic Assay

The 0, 20, 80, and 160 μM NPS-1034-treated BxPC-3 (3000 cells) and MIA PaCa-2 (1000 cells) lines were seeded into a 6-well plate, treated for 1 day, and incubated for 9 days to form colonies. Then, the colonies were fixed with 95% ethanol for 20 min, followed by staining with 20% Giemsa solution at room temperature for 30 min, as described in a previous article [43]. These experiments were conducted in triplicate, and the colony counts of each group were calculated.

### 4.4. Flow Cytometry Analysis

To detect the distribution of the cell cycle and the percentage of apoptotic cells in each group, flow cytometry was performed using a FACSCanto™ II Cell Analyzer (BD Biosciences, Franklin Lakes, NJ, USA), as described in a previous article [44]. We seeded BxPC-3 and MIA PaCa-2 cells into 6-well plates and treated them with 0, 20, and 80 μM NPS-1034 for 48 h. To analyze the distribution of the cell cycle, the cells were fixed with pre-chilled 70% ethanol, resuspended in phosphate-buffered saline (PBS) with 0.4 µg/mL PI and 0.5 mg/mL RNase, and finally determined by flow cytometry. For apoptosis, an Annexin V-FITC apoptosis detection kit (Strong Biotech Corporation, Taipei, Taiwan) was applied under the recommended protocol. After fixation, these cells were resuspended in binding buffer added to 2 μL PI and 2 μL Annexin V-FITC. Then, the cells underwent a reaction in darkness for 15 min and were evaluated for the cell apoptosis rate by flow cytometry. These experiments were conducted in triplicate. The data were analyzed using FlowJ software (BD Biosciences, version 10.10, Franklin Lakes, NJ, USA).

### 4.5. Hoechst Staining Assay

To detect cell apoptosis via morphological changes, Hoechst 33342 staining was conducted. BxPC-3 and MIA PaCa-2 cells were seeded in 6-well plates (2.5 × 10^5^ cells/well), incubated overnight, and treated with NPS-1034 (0, 20, and 80 μM) for 48 h. Next, we fully washed the cells with PBS, stained them with Hoechst 33342 (10 μg/mL, Invitrogen, Waltham, MA, USA), and incubated them for 20 min at 37 °C. We then captured their images with the ImageXpress PICO fluorescence microscope (Molecular Devices, LLC, San Jose, CA, USA), using an excitation wavelength of 350–390 nm and an emission wavelength of 420–480 nm at 20× magnification. Finally, we selected five different fields, counted the condensed nuclei against the total number of nuclei in each field for apoptosis percentages, and plotted the percentages of condensed nuclei for further analysis.

### 4.6. Western Blotting

The BxPC-3 and MIA PaCa-2 cells were treated with 0, 20, and 80 μM NPS-1034 for 24 h and 48 h and harvested. To extract the proteins, the treated cells were lysed in RIPA lysis buffer with complete protease inhibitor cocktail tablets (Roche Applied Science, Mannheim, Germany) dissolved in PBS. We lysed the cells with sonication on ice, centrifuged the extract for 20 min at 4 °C and 13,800× *g*, and stored the supernatant at −80 °C. Later, we measured the protein concentration in the supernatant with a Bio-Rad Protein Assay (Bio-Rad Laboratories, Inc., Contra Costa, CA, USA) to load equal amounts of protein (30 μg) onto sodium dodecyl sulfate polyacrylamide gel electrophoresis (SDS-PAGE) gels. These proteins were then separated by electrophoresis and transferred to ImmobilonTM-P Transfer Membranes (Merck Millipore, Burlington, MA, USA). Next, we blocked the membranes with 5% non-fat milk and incubated them with primary antibodies, including MET (A17366, ABclonal Science, Inc., Woburn, MA, USA), p-MET (Y1349) (AP0077, ABclonal Science, Inc.), PI3K p85 (ab191606, Abcam, plc., Cambridge, UK), p-PI3K (Y607) (ab182651, Abcam, plc.), Akt (ab179463, Abcam, plc.), p-Akt (Ser473) (ab192623, Abcam, plc.), β-catenin (A19657, ABclonal Science, Inc.), E-cadherin (A3044, ABclonal Science, Inc.), N-cadherin (22018-1-AP, Proteintech Group, Inc., San Diego, CA, USA), claudin-1 (13995, Cell Signaling Technology, Inc., Danvers, MA, USA), and actin (AC026, ABclonal Science, Inc.), at 4 °C overnight. We then washed them with Tris-buffered saline containing Tween 20 (TBST) and incubated them again for 1 h with secondary antibodies, including goat anti-rabbit (C04003, Croyez Bioscience Co., Ltd., Taipei City, Taiwan) and goat anti-mouse (C04001, Croyez Bioscience Co., Ltd.). Finally, the membranes were washed with TBST to subsequently react with ImmobilonTM Western Chemiluminescent HRP Substrate (Merck Millipore, Burlington, MA, USA) and were detected for results using the GE Healthcare ImageQuant LAS4000 instrument. The band density was quantified by AlphaEaseFC software (Alpha Innotech, version 3.0, San Leandro, CA, USA), and β-actin was utilized for normalization of the results.

### 4.7. Wound Healing Assays

We performed the wound healing assay with an Ibidi culture insert (Ibidi, Munich, Germany). On each side, 3 × 10^4^ human PDAC cells were seeded and incubated overnight. After removing the insert, we treated the cells with 0, 20, and 80 μM NPS-1034 in serum-free medium and grew them for 16 or 24 h for BxPC-3 cells and 24 or 48 h for MIA PaCa-2 cells. The images were then photographed. These experiments were conducted in triplicate, and the number of migrated cells or the area of each group was calculated using ImageJ software (National Institutes of Health, version 1.52a, Bethesda, MD, USA).

### 4.8. Human Apoptosis Array for Proteome Profiling

The Human Apoptosis Proteome Profiler™ array (R&D Systems, Minneapolis, MN, USA), comprising a nitrocellulose membrane with duplicate spots for 35 apoptosis-related proteins, was applied for proteome profiling. Based on the manufacturer’s protocol, the BxPC-3 and MIA PaCa-2 cells were treated with 80 μM NPS-1034 for 48 h, lysed with 400 μg of protein, and utilized for each array. Membranes were incubated with horseradish peroxidase-conjugated antibody, succeeded with a chemiluminescent detection reagent, and detected with the GE Healthcare ImageQuant LAS4000 (Cytiva, Marlborough, MA, USA). These experiments were conducted in duplicate. ImageJ software was applied to quantify the integrated densities of the spots (National Institutes of Health, version 1.52a, Bethesda, MD, USA).

### 4.9. Next-Generation Sequencing

To further explore clinical applicability, the PDAC cells were treated with commonly prescribed chemotherapy drugs and with or without NPS-1034. The BxPC-3 and MIA PaCa-2 cells were treated with 20 μM fluorouracil, 10 μM oxaliplatin, 20 μM fluorouracil + 50 μM NPS-1034, and 10 μM oxaliplatin + 50 μM NPS-1034 for 24 h. Their total RNA was extracted with TRIzol^®^ Reagent (Invitrogen, USA) in accordance with the instruction manual. Genomics (Taiwan) performed sample preparation, library preparation, sequencing, alignment, and differential expression analysis. The analyses were then carried out according to official protocols. The bases with low quality and sequences from adapters in raw data were removed using the program fastp (version 0.20.0). The filtered reads were aligned to the reference genomes using HISAT2 (version 2.1.0). The software FeatureCounts (v2.0.1) in the Subread package was applied for quantification of the gene abundance. Differentially expressed genes (DEGs) were identified by EdgeR (version 3.36.0). In the pooling data, genes with a *p*-value ≤ 0.05 were considered significantly differentially expressed. Gene Ontology (GO) analysis for overrepresented biological processes and Kyoto Encyclopedia of Genes and Genomes (KEGG) pathway analysis for enrichment were conducted using the compare cluster function in the R package clusterProfiler (version 4.7.1). The top eight GO terms and the top five KEGG pathways that accounted for the highest proportions of genes with significantly differential expression were visualized with a cut-off criterion of *p* < 0.05. The original data are available upon request from the corresponding author.

### 4.10. Statistical Analysis

IBM SPSS software (version 20.0) (Armonk, NY, USA) was utilized for the statistical analysis. Data are demonstrated as mean ± standard deviation (SD). For continuous or discrete data analysis, Student’s *t*-test was applied, as described in [43,45]. Statistical tests were conducted as a two-sided model, and values of *p* < 0.05 were considered statistically significant (* *p* < 0.05; ** *p* < 0.01; *** *p* < 0.001).

## 5. Conclusions

The present study investigated the influence of NPS-1034, a dual inhibitor of MET and AXL, on PDAC cell lines. For monotherapy, a 24-hour treatment inhibited migration through the MET-induced EMT pathway, and a 48-hour treatment inhibited apoptosis significantly. Dual therapy in combination with fluorouracil/oxaliplatin not only showed a synergistic effect, significant decrease in viability, and increase in apoptosis but also showed IFN and TNF induction, which may be the reason for the apoptosis. This is the first study of NPS-1034 and pancreatic cancer and the first to reveal the immunoregulatory properties of NPS-1034. As a multi-RTK inhibitor and immunoregulatory agent, NPS-1034 could be highly beneficial for PDAC patients. Therefore, further in vivo studies are needed.

## Data Availability

Data is contained within the article or Supplementary Materials.

## Supplementary Materials

The following supporting information can be downloaded at: https://www.mdpi.com/article/10.3390/ijms25136919/s1.
