# Supplementary material for: The Therapeutic Role of NPS-1034 in Pancreatic Ductal Adenocarcinoma as Monotherapy and in Combination with Chemotherapy
Source: Int J Mol Sci. 2024 Jun 24;25(13):6919. doi: 10.3390/ijms25136919 (PMC11241054; doi:10.3390/ijms25136919)
Supplement: Supplementary file 1 [file ijms-25-06919-s001.zip › ijms-3040008-supplementary.pdf]

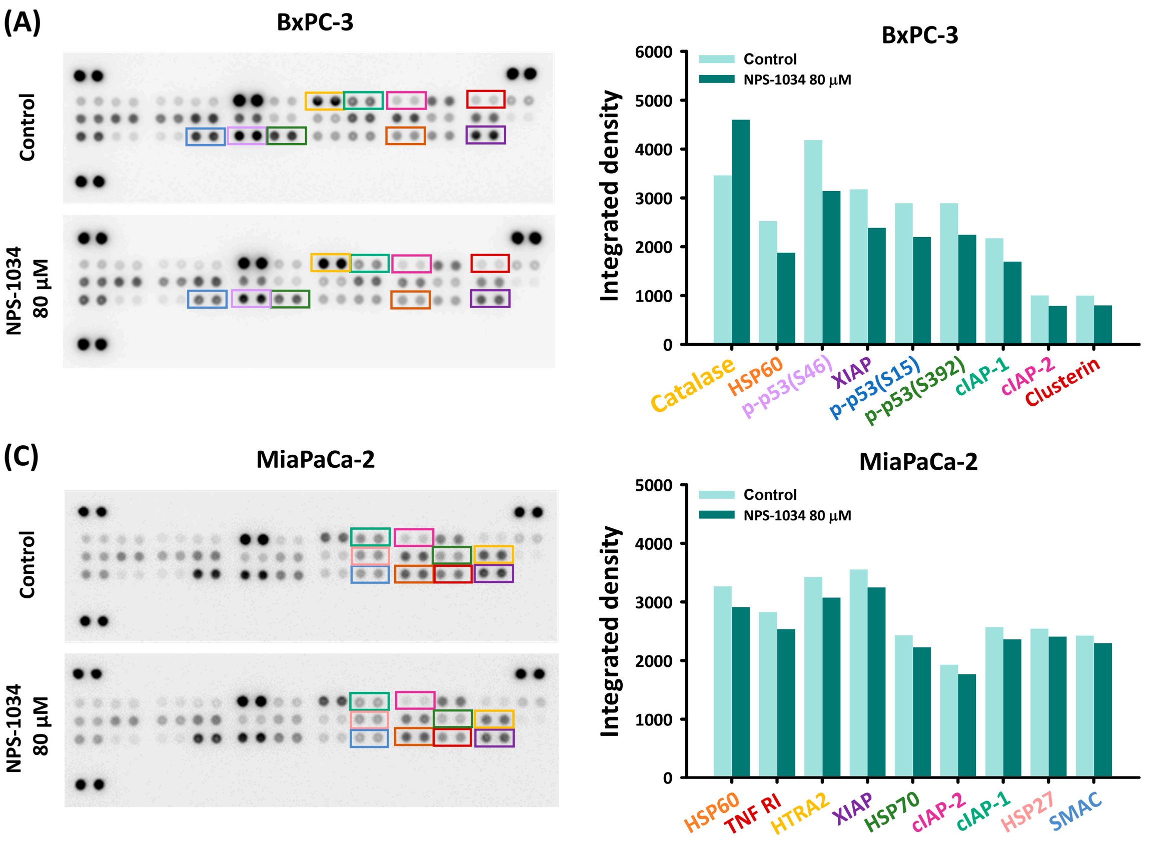

Supplementary Figure S1. Determination of apoptosis-related protein expression altered by NPS-1034 treatment between PDAC cells.

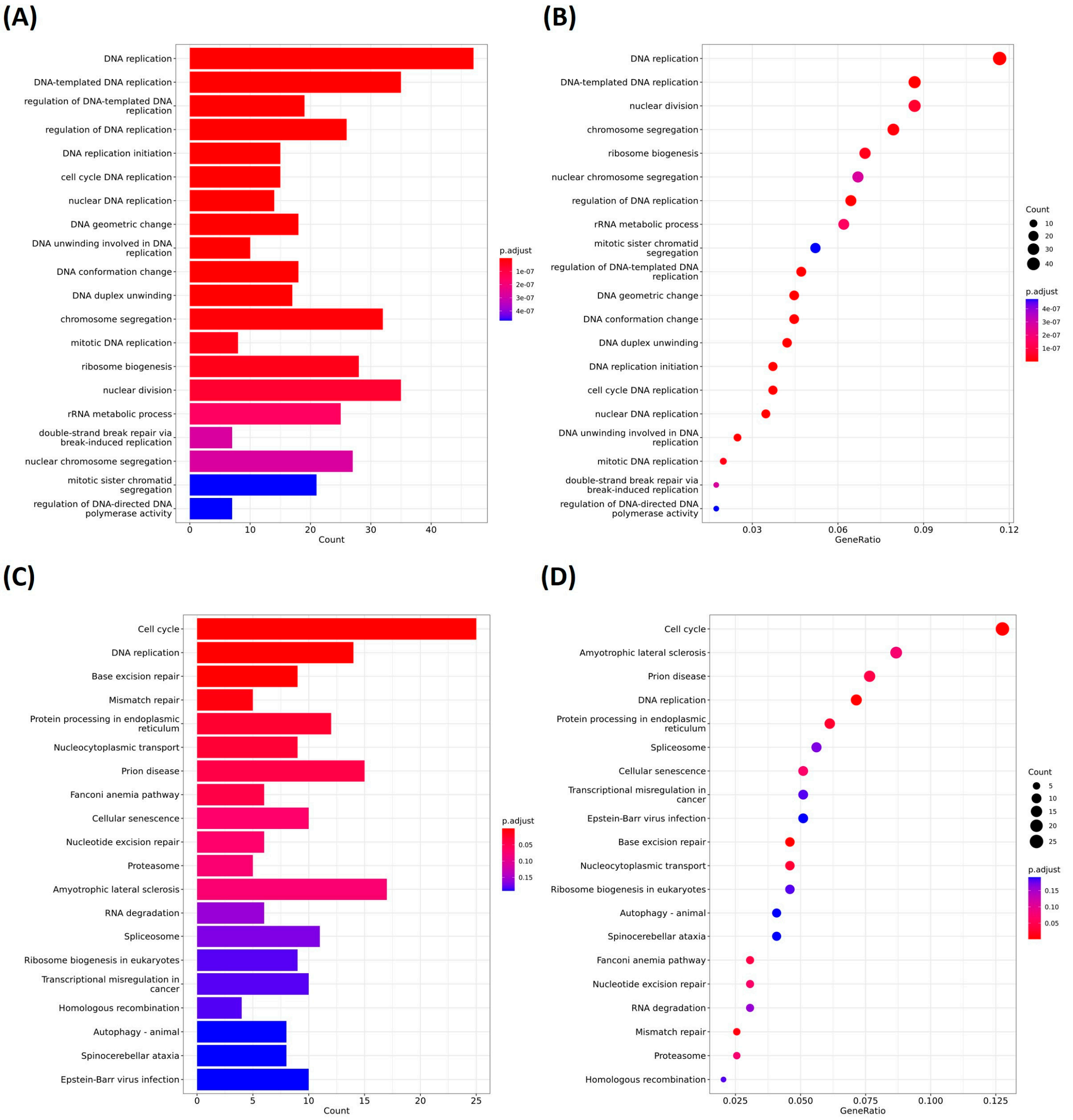

Supplementary Figure S2. NGS analysis using the GO and KEGG databases revealed changes in gene expression between PDAC cells treated with and without NPS-1034. Data from both cell lines were combined for this analysis. (A,B) Results of the control group compared to NPS-1034 treatment using the GO database. (C,D) Results of the control group compared to NPS-1034 treatment using the KEGG database. (A,C) The counts of significantly changed genes in each group are shown, with groups ordered by p-value. (B,D) The ratios of significantly changed genes in each group are shown, with groups ordered by the change ratio.
